# An ultrasensitive label-free electrochemical immunosensor based on signal amplification strategy of multifunctional magnetic graphene loaded with cadmium ions

**DOI:** 10.1038/srep21281

**Published:** 2016-02-16

**Authors:** Faying Li, Yueyun Li, Yunhui Dong, Liping Jiang, Ping Wang, Qing Liu, Hui Liu, Qin Wei

**Affiliations:** 1 School of Chemical Engineering, Shandong University of Technology, Zibo, 255049, P.R. China; 2 Key Laboratory of Chemical Sensing & Analysis in Universities of Shandong, School of Chemistry and Chemical Engineering, University of Jinan, Jinan, 250022, P.R. China

## Abstract

Herein, a novel and ultrasensitive label-free electrochemical immunosensor was proposed for quantitative detection of human Immunoglobulin G (IgG). The amino functionalized magnetic graphenes nanocomposites (NH_2_*-*GS*-*Fe_3_O_4_) were prepared to bond gold and silver core-shell nanoparticles (Au@Ag NPs) by constructing stable Au*-*N and Ag*-*N bond between Au@Ag NPs and -NH_2_. Subsequently, the Au@Ag/GS*-*Fe_3_O_4_ was applied to absorb cadmium ion (Cd^2+^) due to the large surface area, high conductivity and exceptional adsorption capability. The functional nanocomposites of gold and silver core-shell magnetic graphene loaded with cadmium ion (Au@Ag/GS*-*Fe_3_O_4_/Cd^2+^) can not only increase the electrocatalytic activity towards hydrogen peroxide (H_2_O_2_) but also improve the effective immobilization of antibodies because of synergistic effect presented in Au@Ag/GS*-*Fe_3_O_4_/Cd^2+^, which greatly extended the scope of detection. Under the optimal conditions, the proposed immunosensor was used for the detection of IgG with good linear relation in the range from 5 fg/mL to 50 ng/mL with a low detection limit of 2 fg/mL (S/N = 3). Furthermore, the proposed immunosensor showed high sensitivity, special selectivity and long-term stability, which had promising application in bioassay analysis.

Human Immunoglobulin G (IgG) is an important component in the immune system, which plays a crucial role in recognizing bacteria and viruses^1^,^2^. In addition, it has also been administered therapeutically from exogenous pooled donor sources^3^,^4^. The concentration of IgG in blood and other body fluid is direct correlation to the standard of humoral immunity, which abnormal IgG concentrations often indict the risk of disease or vulnerability to infection^3^,^5^. Therefore, it is necessary to develop method for quantitative detection IgG for immunological diagnosis.

In recent years, various detection assays have been developed for the detection of IgG, such as chemiluminescence^6^, colorimetric sensors^7^,^8^, fluorescence^9^,^10^ and electrochemical immunosensors^11^,^12^. In comparisons, electrochemical immunoassay may be an excellent candidate for the detection of tumor markers because of fast analytical time, low detection limits, high sensitivity, simple pretreatment procedure and inexpensive instrumentation^13^,^14^. As one important branch of electrochemical immunosensors, the label-free immunosensors have became an attractive and promising approach to direct detection of tumor makers because simple procedure, ease of use and rapidity of the assay and without the use of secondary antibody compared with sandwich-type immunosensors^15^,^16^.

Over the past decade, a wide variety of nanomaterials have been designed as transducing materials to fabricate label-free electrochemical immunosensors, such as, graphene sheets (GS)^17^,^18^, metal oxides^19^,^20^ and noble metal nanoparticles^21^,^22^. As a kind of popular transducing materials, graphene oxide (GO) possess many oxygen-containing functional groups which make the water-solubility better and make it easy to form a stable chemical bond with various materials, such as, magnetic nanostructures, metallic or catalytic^23^,^24^. To date, Fe_3_O_4_ NPs have attracted a considerable interest due to its good biocompatibility^25^ and great auxiliary catalytic activity toward the reduction of hydrogen peroxide (H_2_O_2_)^20^,^26^. Therefore, the graphene was introduced to combine with Fe_3_O_4_ NPs by chemical reaction. Among the noble metal nanoparticles, gold nanoparticles (Au NPs) and silver nanoparticles (Ag NPs) have attracted a considerable interest because of its good biocompatibility^27^ and superior auxiliary catalytic activity towards the reduction of H_2_O_2_^28^,^29^. Further, Au and Ag NPs can facilitate the electron transfer because of its superior electrochemical properties. So, Au NPs are widely used in fixing antibody because of its superior biocompatibility and chemical stability^30^,^31^. Compared with Au NPs, Ag NPs exhibit excellent electro catalytic activity towards the reduction of H_2_O_2_^32^,^33^. Compared with single metal NPs, bimetallic NPs with a core–shell structure show distinctly unique characteristics than their monometallic counterparts^34^. In addition, the Au@Ag NPs could also enable the facile conjugation of capture antibodies because of the stable Au-N^35^,^36^ and Ag-N^37^ bond between Au@Ag NPs and –NH_2_ on antibodies. Simultaneously, the amino functionalized magnetic graphenes nanocomposites (NH_2_*-*GS*-*Fe_3_O_4_) was applied to absorb Cd^2+^ 38,39 due to the large surface area, high conductivity and exceptional adsorption capability. The adsorbed Cd^2+^, can further promote the redox of H_2_O_2_, which was applied to signal amplification. The signal amplification strategy, using the synergetic effect present in functional nanocomposites of gold and silver core-shell magnetic graphene loaded with cadmium ion (Au@Ag/GS*-*Fe_3_O_4_/Cd^2+^), can further increase electron transfer efficiency on electrode surface and the reaction efficiency of the nanocomposite toward H_2_O_2_ reduction to improve the detection sensitivity of the immunosensor.

In this research, a novel and ultrasensitive label-free immunosensor for the quantitative detection the IgG was prepared using Au@Ag/GS*-*Fe_3_O_4_/Cd^2+^ as a signal amplification platform. The synergic effect between functionalized magnetic graphene nanocomposites (GS-Fe_3_O_4_), Au@Ag NPs and Cd^2+^ can not only increase the electrocatalytic activity towards hydrogen peroxide (H_2_O_2_) but also improve the effective immobilization of antibodies, which greatly extend the scope of detection. The proposed immunosensor provides a useful technology for the quantitative detection of IgG in human serum, shows high sensitivity, good selectivity and stability for the quantitative detection of IgG, holding a great potential in clinical and diagnostic applications.

## Experimental

### Apparatus and reagents

Scanning electron microscopy (SEM) images and energy-dispersive X-ray spectroscopy (EDS) analysis were collected using a FEI QUANTA FEG250 coupled with INCA Energy X-MAX-50. Fourier transform infrared spectroscopy (FTIR) spectrum was collected using VERTEX 70 spectrometer (Bruker, Germany). All electrochemistry measurements were performed on a CHI760E electrochemical workstation (Chenhua Instrument Shanghai Co., Ltd, China) by using a conventional three-electrode system consisted of a glassy carbon electrode (GCE, 4 mm in diameter) as working electrode, a saturated calomel electrode (SCE) as the reference electrode, and the platinum wire electrode as the counter electrode.

HAuCl_4_·4H_2_O was purchased from Sinopharm Chemical Reagent Co., Ltd (Shanghai, China). FeCl_3_∙6H_2_O was purchased from Damao Chemical Reagent Co., Ltd (Tianjin, China). 3-aminopropyl triethoxysilane (APTES) was purchased from XiBao biological technology co., Ltd. (Shanghai, China). Human immunoglobulin G (IgG), goat Anti-Human IgG and bovine serum albumin (BSA, 96–99%) were obtained from Dingguo Biochemical Reagents (Beijing, China). All other reagents were at analytical grade and ultrapure water was used throughout the study.

### Preparation of the NH_2_- GS−Fe_3_O_4_

Graphene oxide (GO) was synthesized according an improved Hummer’s method^40^. In brief, graphite flakes (0.6 g) and KMnO_4_ (3.6 g) were dispersed in a mixture of concentrated H_2_SO_4_ (72 mL) and H_3_PO_4_ (8 mL). Subsequently, the reaction was heated to 50 °C and maintained at this temperature for 12 h with stirred. The mixture was cooled to room temperature after reaction and poured onto ice (80 mL) with 30% H_2_O_2_ (0.6 mL). Then, the mixture was centrifuged and removed the supernatant. After that, the remaining solid material was thoroughly washed with water, 0.2 mol/L HCl (60 mL), ethanol and ether. Finally, the obtained solid material was dried in vacuum overnight.

GS−Fe_3_O_4_ was synthesized according to a protocol described previously^39^. FeCl_3_·6H_2_O (0.5 g) was dissolved in ethylene glycol (10 mL) to form a clear solution, then, NaAc (1.5 g), ethanediamine (5 mL) and GO (0.5 g) was added into the mixture orderly and dissolved under stirred vigorously for 30 min. Subsequently, the mixture was transferred into the teflon-lined stainless steel autoclave. The autoclave was heated to and maintained at 200 °C for 8 h and cooled down to room temperature after reaction. The prepared compound sample was thoroughly washed to remove the impurities and separated via a strong magnet. The resulting GS−Fe_3_O_4_ was dried under high vacuum overnight. It should be noted that the GO was translated into the graphene sheet (GS) in the process of reaction.

The amino-functionalized GS-Fe_3_O_4_ (NH_2_-GS-Fe_3_O_4_) was synthesized by an improved method^41^. Briefly, GS-Fe_3_O_4_ (0.1 g) was dispersed in a solution of ethanol (10 mL) containing APTES (0.1 mL). Subsequently, the solution was heated to 70 °C and kept for 1.5 h. Finally, the NH_2_-GS-Fe_3_O_4_ was obtained by magnetic separation and dried under high vacuum overnight.

### Preparation of Au@Ag/GS-Fe_3_O_4_

The preparation of Au NPs was referred to the classical Frens method^42^. In brief, Sodium citrate (1.5 mL, 10 mg/mL) was added to the aqueous solution (100 mL) containing HAuCl_4_ (1 wt%, 1 mL). Then, the mixture was heated to reflux and kept for 15 min. A wine red solution of Au NPs was obtained after being cooled to room temperature and stored at 4 °C. Au@Ag NPs was synthesized according an to the literature previously^43^. 1.0 mL of ascorbic acid (100 mmol/L) and 0.5 mL of AgNO_3_ (10 mmol/L) were added into 20 mL of hexadecyl trimethyl ammonium bromide (CTAB) solution (50 mmol/L). After that, 2 mL of the Au NPs solution was added into the mixed solution. Then, 0.1 mL of NaOH aqueous solution (1 mol/L) was added drop-wise to the above solution under stirred vigorously. In this way, the color of the solution changed from red to bright golden yellow, indicating the success preparation of Au@Ag NPs.

The prepared NH_2_-GS-Fe_3_O_4_ (10 mg) was dispersed in the Au@Ag NPs (40 mL) solution. The suspension was stirred for 24 h and magnetic separation. Au@Ag NPs could bind with amino groups on the surface of NH_2_-GS-Fe_3_O_4_. The sediment was dried and the obtained powder was designated as Au@Ag/GS-Fe_3_O_4_.

### Preparation of Au@Ag/GS-Fe_3_O_4_/Cd^2+^

**Figure 1:**
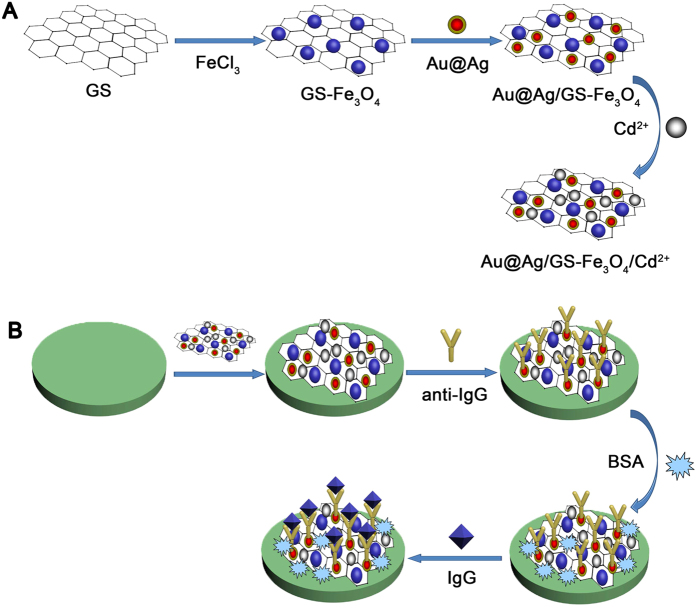
(**A**) The prepare process of Au@Ag/GS-Fe_3_O_4_/Cd^2+^; (**B**) Schematic diagram for fabrication of the label-free electrochemical immunosensor.

Au@Ag/GS-Fe_3_O_4_ (10 mg) was dispersed into cadmium sulphate solution (10 mL, 2 mg/mL). The solution had been oscillated for 24 h to ensure that Cd^2+^ could be fully absorbed onto the Au@Ag/GS-Fe_3_O_4_. The Au@Ag/GS-Fe_3_O_4_/Cd^2+^ was obtained for further use after magnetic separation. Figure 1A shows the preparation process of the Au@Ag/GS-Fe_3_O_4_/Cd^2+^.

### Fabrication of the immunosensor

The schematic diagram of the stepwise self-assembly procedure of the proposed label-free immunosensor is shown in Fig. 1B. Generally, GCE was polished to a mirror-like, and washed thoroughly with ultrapure water. Firstly, the aqueous solution of Au@Ag/GS-Fe_3_O_4_/Cd^2+^ (2 mg/mL, 6 μL) was coated onto the surface of GCE and dried at room temperature. After drying for 1 h, the resultant Au@Ag/GS-Fe_3_O_4_/Cd^2+^/GCE was incubated with anti-IgG (10 μg/mL, 6 μL) by the chemical bonding between Au@Ag NPs and available amine groups of anti-IgG. After incubated for another 1 h at 4 °C, BSA solution (1 wt%, 3 μL) was added onto the electrode to eliminate nonspecific binding sites. After 1 h incubation, the BSA/anti-IgG/Au@Ag/GS-Fe_3_O_4_/Cd^2+^/GCE was washed with ultrapure water and incubated with a varying concentration of IgG (5 fg/mL to 50 ng/mL, 6 mL) for 1 h at room temperature, and then the IgG/BSA/anti-IgG/Au@Ag/GS-Fe_3_O_4_/Cd^2+^/GCE was washed extensively to remove unbounded IgG molecules. Ultimately, the proposed immunosensor was stored at 4 °C for further usage.

### Detection of IgG

Phosphate buffered saline (PBS, pH = 6.8) were prepared by mixing Na_2_HPO_4_ and KH_2_PO_4_ stock solution and used as the electrolyte in the process of electrochemical measurements. Amperometric i–t curve was used to record the amperometric response by scanning the potential at −0.4 V. 5 mM H_2_O_2_ was added into the PBS (10 mL) after the back ground current was stabilized. The cyclic voltammetry (CV) experiments were recorded in 5 mM K_3_[Fe(CN)_6_] by scanning the potential from −1 V to 1 V. For A.C. impedance measurements, a frequency range of 0.1 kHz to 100 Hz and AC voltage amplitude of 5 mV were used.

## Results and Discussion

### Characterization of Au/Ag@ GS-Fe_3_O_4_

**Figure 2:**
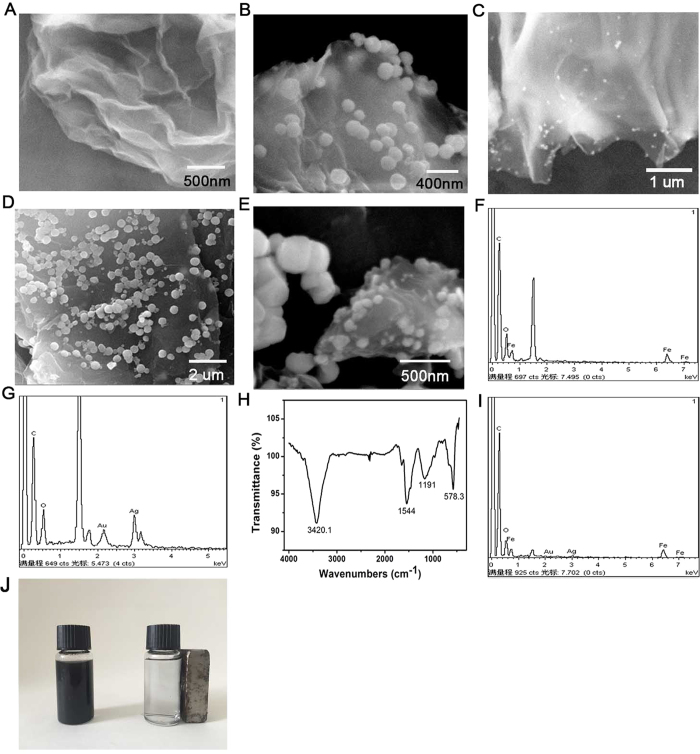
SEM image: GO (**A**), GS-Fe_3_O_4_ (**B**); Au@Ag/GS (**C**); Au@Ag/GS-Fe_3_O_4_ (**D**,**E**). EDX spectrum: GS-Fe_3_O_4_ (**F**); EDX spectrum of the Au@Ag/GS (**G**); FT-IR spectrometer analysis of GS-Fe_3_O_4_ (**H**); comparison of Au@Ag/GS-Fe_3_O_4_ in the absence and presence of a magnet (**I**).

As shown in (Fig. 2A), the surface of GO exhibits wrinkled, paper-like structure. After magnetization, GS was loaded with a lot of nearly monodisperse microspheres Fe_3_O_4_ (Fig. 2B). It was observed that these Fe_3_O_4_ NPs own quasi-monodisperse size with an average grain diameter of 230 nm by the SEM. Obvious Fe, C, and O elements were observed which prove Fe_3_O_4_ was loaded on the GS successfully (Fig. 2F). Furthermore, FT-IR spectra of GS-Fe_3_O_4_ were recorded to prove that GS was loaded successfully with Fe_3_O_4_ (Fig. 2H). As shown in Fig. 2G, the peak at 3420 cm^−1^ corresponding to the hydroxyl group stretching vibration (O–H), two peaks at 1544 and 1191 cm^−1^ corresponded to the carboxyl (–COOH) and carbonyl (C=O) stretching vibrations. A peak at 573 cm^−1^ was typical Fe–O stretching vibration of the prepared GS-Fe_3_O_4_ composites^44^. This result indicted that Fe_3_O_4_ NPs was loaded successfully on the surface of GO. When the Au@Ag NPs were coated on the surface of NH_2_-GS, the surface morphology was greatly alternated. Au@Ag NPs are well monodispersed and uniformly spherical in shape. As shown in (Fig. 2C), many small particles were loaded on the NH_2_-GS by constructing stable Au-N and Ag-N bond between Au@Ag NPs and -NH_2_. Subsequently, EDX spectrum of Au@Ag-GS was recorded which clearly confirmed the presence of Au@Ag NPs attached on the surface of the NH_2_-GS. As shown in (Fig. 2G), obvious Au, Ag, C, and O elements were observed, in which the signal of Au, Ag are assigned to the Au@Ag NPs, and that of C and O elements are belonged to the GS. Figure 2D,E shows the SEM image of Au@Ag/GS-Fe_3_O_4_ in different scale respectively, which contains two kinds of size of particle. It is the combination of the Fig. 2B,C, suggesting the synthesized of Au@Ag/GS-Fe_3_O_4_ successfully. Furthermore, obvious Au, Ag, C, and O and Fe elements were observed in Fig. 2I, which also suggest the synthesized of Au@Ag/GS-Fe_3_O_4_ successfully. Figure 2J also proved that the magnetic field presents significant effect on the dispersion.

### The mechanism of multiple signal amplification strategy

**Figure 3:**
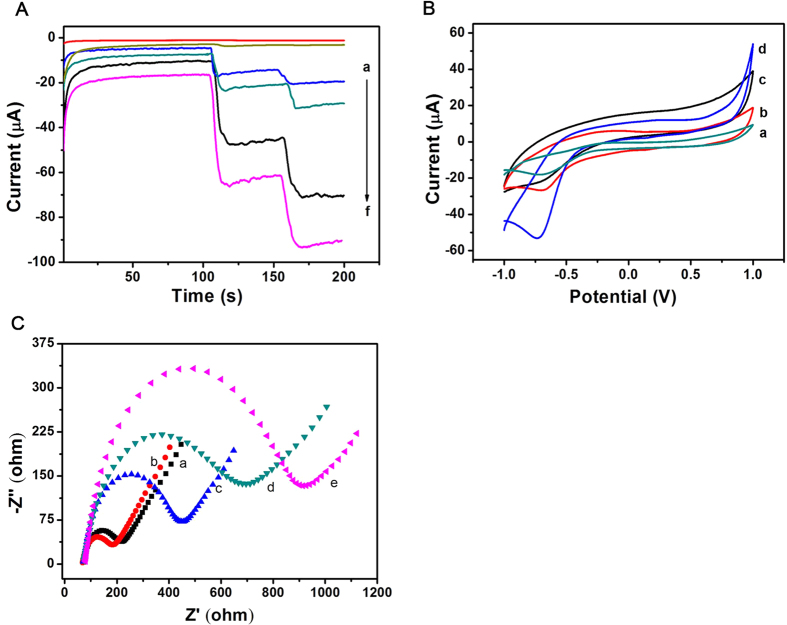
(**A**) Ameperometic response: (a) bare GCE, (b) GO, (c) GS-Fe_3_O_4_, (d) Au@Ag NPs, (e) Au@Ag/GS-Fe_3_O_4_, (f) Au@Ag/GS-Fe_3_O_4_/Cd^2+^; (**B**) CV of the immunosensor: a bare GCE was scanned in 0.1 mg/mL of Cd^2+^ from −1.0 V to 1.0 V (a); using Au@Ag/GS-Fe_3_O_4_ as signal amplification platform (c); using Au@Ag/GS-Fe_3_O_4_/Cd^2+^ as signal amplification platform in PBS at pH = 6.8 before (b) and after (d) the addition of 5 mM H_2_O_2_; (**C**) Nyquist plots of the EIS method: GCE (a), Au@Ag/GS-Fe_3_O_4_/Cd^2+^/GCE (b), anti-IgG/Au@Ag/GS-Fe_3_O_4_/Cd^2+^/GCE (c), BSA/anti-IgG/Au@Ag/GS-Fe_3_O_4_/Cd^2+^/GCE (d), IgG/BSA/anti-IgG/Au@Ag/GS- Fe_3_O_4_/Cd^2+^/GCE (**e**).

The sensitivity of the label-free immunosensor was affected directly by the superior electrocatalytic properties of Au@Ag/GS-Fe_3_O_4_/Cd^2+^ towards the reduction of H_2_O_2_. To comparative analysis, the amperometric i-t curve was employed to record the catalytic properties of bare GCE, GO, GS-Fe_3_O_4_, Au@Ag NPs, Au@Ag/GS-Fe_3_O_4_, Au@Ag/GS-Fe_3_O_4_/Cd^2+^ to H_2_O_2_. As shown in Fig. 3A, there no catalytic effect for bare GCE towards the reduction of H_2_O_2_ (curve a). In addition, a very weak signal was detected when GS (curve b) were loaded on the electrode. Fe_3_O_4_ possess excellent auxiliary catalytic activity towards the reduction of H_2_O_2_^20^. When GS-Fe_3_O_4_ was applied as the signal amplification platform, the current response was significantly increased (curve c). Simultaneously, a much larger current response was observed when Au@Ag NPs was applied to modify the bare GCE due to the synergistic effect of Au NPs^28^ and Ag NPs^45^ towards the reduction of H_2_O_2_ (curve d). Subsequently, the current response was further increased (curve e) when the electrode was modified with Au/Ag@GS-Fe_3_O_4_. As expected, using Au@Ag/GS-Fe_3_O_4_/Cd^2+^ as signal amplification platform displayed the highest current change due to synergistic effect (curve f). The result indicted that Fe_3_O_4_ NPs, Au@Ag NPs and Cd^2+^ promote the multiple signal amplification toward the reduction of H_2_O_2_ as an analytical signal. Hence, the resultant nanocomposites (Au@Ag/GS-Fe_3_O_4_/Cd^2+^) was adopted as signal amplification platform due to excellent electrochemical performance to improve the sensitivity.

CV was applied to further verify that the Cd^2+^ was adsorbed successfully onto the Au@Ag/GS-Fe_3_O_4_ and have better electrocatalytic properties toward the reduction of H_2_O_2_ (Fig. 3B). A reduction peak at −0.7 V is obvious when a bare GCE was scanned in 0.1 mg/mL of Cd^2+^ solution (curve a). Simultaneously, a reduction peak (curve b) was also observed at −0.7 V when a bare GCE was scanned in PBS (pH = 6.8) using Au@Ag/GS-Fe_3_O_4_/Cd^2+^ as signal amplification platform. Hence, it inferred that Cd^2+^ was adsorbed successfully onto the Au@Ag/GS-Fe_3_O_4_. Furthermore, there was no current response (curve c) when the electrode was scanned in PBS (pH = 6.8) using Au@Ag/GS-Fe_3_O_4_ as signal amplification platform. The comparison clearly suggests that the observed current response was directly associated with absorbed Cd^2+^. In order to verify that Cd^2+^ possess better electrocatalytic properties toward the reduction of H_2_O_2_. As shown in Fig. 3B, a bare GCE was modified with Au@Ag/GS-Fe_3_O_4_/Cd^2+^ in PBS (pH = 6.8) with the addition of 5 mM H_2_O_2_. After the addition of H_2_O_2_, a dramatic increase of the reduction current (curve d) was observed at −0.7 V, which further verify the Au@Ag/GS-Fe_3_O_4_/Cd^2+^ has a good electrocatalytic performance towards the reduction of H_2_O_2_.

### Electrochemical impedance spectroscopy (EIS) characterization of immunosensor

Electrochemical impedance spectroscopy (EIS) was regarded as an effective method to characterize the fabrication process of the proposed immunosensor by monitoring the interfacial properties^46^ and the changes of the electron-transfer resistance (Ret)^47^. The Nyquist plots of EIS were recorded in a solution which consist of 0.1 M KCl and 2.5 mmol/L Fe(CN)_6_^3^−/Fe(CN)_6_^4^−, the frequencies from 0.1 to 10^5 ^Hz and the potentiostatic at 0.188 V.

As shown in Fig. 3, the bare GCE exhibited a smaller Ret (curve a). After the Au@Ag/GS-Fe_3_O_4_/Cd^2+^ was modified on the electrode, the semicircle is much smaller (curve b) due to the high electrical transport properties of Au@Ag/GS-Fe_3_O_4_/Cd^2+^. After incubation with anti-IgG, the Ret (curve c) was significantly increased due to the anti-IgG is protein which can hinder electron transfer, which indicates anti-IgG was immobilized on the electrode successfully. Followed by blocking the nonspecific binding spots with BSA, the Ret (curve d) was further increased due to the blocking effect on electron transferring by the modified protein molecules on the surface of the electrode. Additionally, the Ret further increased with the addition of IgG (curve e), which indicates the successful capture of IgG and the formation of immunocomplex layer hinder the electron transfer. As a result, we can conclude that the proposed immunosensor was fabricated successfully.

### Optimization of experimental conditions

**Figure 4:**
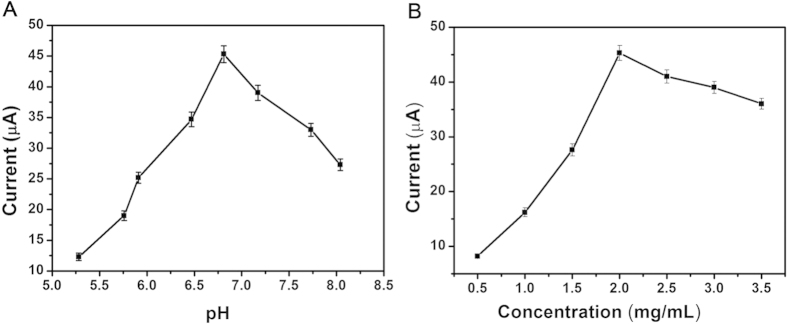
(**A**) The optimization of experimental conditions with pH, (**B**) Au@Ag/GS-Fe_3_O_4_/Cd^2+^ concentration, Error bar = RSD (n = 5).

In order to detect optimal electrocatalytic signal, it was necessary to optimize the experimental conditions including pH and the concentration of Au@Ag/GS-Fe_3_O_4_/Cd^2+^. The pH of the PBS was investigated with same concentration of Au@Ag/GS-Fe_3_O_4_/Cd^2+^ (2.0 mg/mL). As shown in Fig. 4A, the current signal increases with the variation of pH from 5.6 to 6.8, and then decreases with the variation of pH from 6.8 to 8.1. The optimal amperometric response was obtained at pH = 6.8. It was inferred that the pH value obviously affected the electrocatalytic process of Au@Ag/GS-Fe_3_O_4_/Cd^2+^ toward the reduction of H_2_O_2_. Simultaneously, the highly acidic or alkaline surroundings would influence the activity of the antigens, antibodies and damage the immobilized protein^48^,^49^. By contrast, the antigens and antibodies could keep their bioactivity in this approximate neutral conditions of pH^50^.

The concentration of Au@Ag/GS-Fe_3_O_4_/Cd^2+^ would affect the amperometric response by accelerateing the electron transfer efficiency and immobilize of antibodies. As shown in Fig. 4B, with the increasing of the concentration from 0.5 mg/mL to 2.0 mg/mL, the current signal increased, but the current signal decreased with the concentration from 2.0 mg/mL to 3.5 mg/mL. The optimal amperometric response was obtained at 2.0 mg/mL. It was inferred that the increasing of Au@Ag/GS-Fe_3_O_4_/Cd^2+^ film thickness might lead to an increase of interface electron transfer resistance, and the electron transfer become more difficult. Simultaneously, the higher or lower concentrations of Au@Ag/GS-Fe_3_O_4_/Cd^2+^ influenced the catalytic performance for the reduction of H_2_O_2_^51^. Therefore, the concentration of 2.0 mg/mL was used as the optimal concentration for this study.

In addition, the rest experiment conditions were also required strictly. For example, the concentration of antibodies was 10 μg/mL, the incubation time of 1 h, which was enough to capture antigen (ng/mL) and achieve the specific recognition between antigens and antibodies^52^. Under the optimal conditions, the proposed immunosensor will obtain an optimal electrochemical response for quantitative detection of IgG.

### Analytical performance of the immunosensor

**Figure 5:**
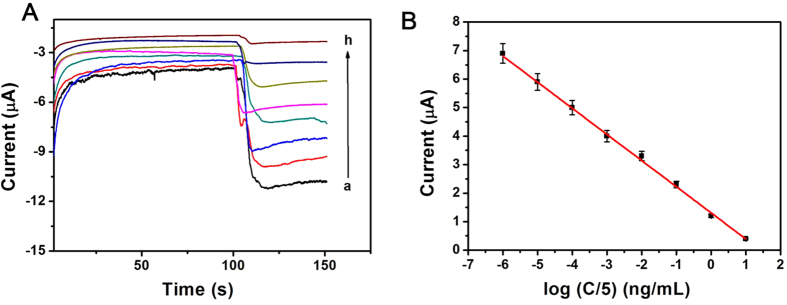
(**A**) Amperometric response of the immunosensor to different concentration of IgG, from a to h: 5 fg/mL, 50 fg/mL, 500 fg/mL, 5 pg/mL, 50 pg/mL, 500 pg/mL, 5 ng/mL, 50 ng/mL; (**B**) Calibration curves of immunosensor to different concentrations of IgG, error bar = RSD (n = 5).

Under the optimal experimental conditions, amperometric i-t curve was used to detect different concentrations of IgG (Fig. 5A) using Au@Ag/GS-Fe_3_O_4_/Cd^2+^ as a signal amplification platform in pH 6.8 PBS. The amperometric response towards the reduction of 5 mmol/L H_2_O_2_ and IgG concentrations were shown in Fig. 5A. As can be seen, the i-t current response change was linearly related to the logarithmic values of the IgG concentration within the range from 5 fg/mL to 50 ng/mL, and with an extremely low limit of detection of 2 fg/mL (S/N = 3). The regression equation of the calibration curve as: I = 1.301−0.9168 logC with correlation coefficient of 0.9970 (Fig. 5B). The results demonstrate the proposed method have an acceptable quantitative performance for the IgG detection. The performance of the immunosensor was compared with previously reported methods for the detection of IgG. As shown in Supplementary Table S1, the proposed immunosensor has a wider linear range and lower detection limit than previously report. The low detection limit was attributed to several factors. Firstly, Au@Ag/GS-Fe_3_O_4_/Cd^2+^, a novel material, is a combination of Fe_3_O_4_-GS, Au@Ag NPs and Cd^2+^ through covalent bonding, which has high electrocatalytic performance towards the reduction of H_2_O_2_ leading to higher sensitivity. Secondly, Au@Ag NPs not only possess good biocompatibility which firmly conjugated with a relatively large amount of antibodies, but also in assisting the electron transfer process to amplify the signal. In addition, Cd^2+^ possess good electrocatalytic activity towards the reduction of H_2_O_2_, which can be used to amplify the detection signal and leaded to the high sensitivity of the designed immunosensor. Consequently, the synergic effect between Au@Ag/GS-Fe_3_O_4_/Cd^2+^ can not only increase the electrocatalytic activity towards H_2_O_2_ but also improve the effective immobilization of antibodies, which could greatly improve the probability of antibody–antigen recognition and extended the scope of detection. Hence, high sensitivity is one of the characteristics of the proposed immunosensor.

### Reproducibility, selectivity and stability

**Figure 6:**
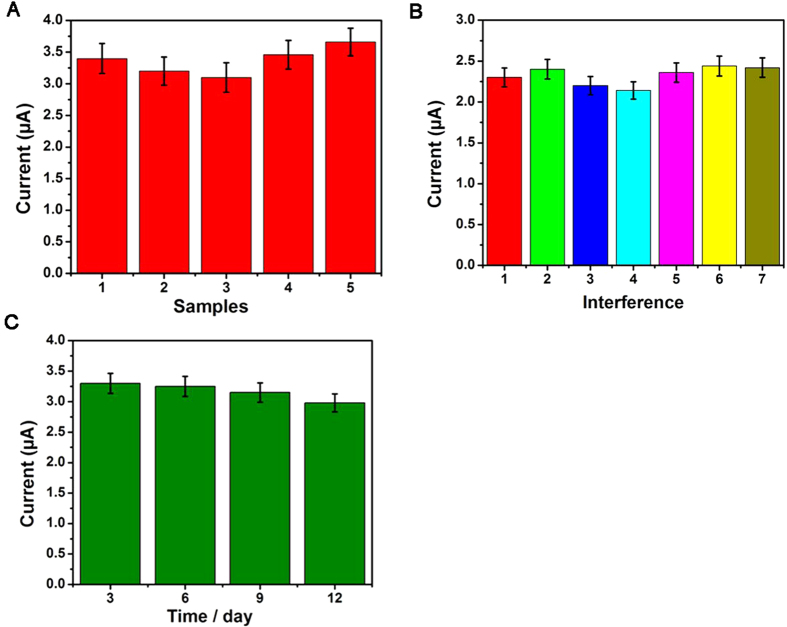
(**A**) Amperometric change response of biosensor to different electrodes treated in same way; (**B**) Current responses of the immunosensor to 0.5 ng/mL IgG (1), 0.5 ng/mL IgG +50 ng/mL AFP (2), 0.5 ng/mL IgG +50 ng/mL BSA (3), 0.5 ng/mL IgG +50 ng/mL CEA (4), 0.5 ng/mL IgG +50 ng/mL glucose (5), 0.5 ng/mL IgG + 50 ng/mL BSA +50 ng/mL AFP (6), 0.5 ng/mL IgG +50 ng/mL BSA +50 ng/mL CEA (7). Error bar = RSD (n = 5).

To evaluate the reproducibility of the immunosensor, a series of five different electrodes were prepared for the detection of 0.05 ng/mL of IgG (Fig. 6A). The relative standard deviation (RSD) of the measurements for the five electrodes was 3.6%, indicating the precision and reproducibility of the immunosensor is acceptable.

To investigate the selective of the proposed immunosensor, interferences study was performed using (alpha fetoprotein) AFP, BSA, carcinoembryonic antigen (CEA), and glucose. IgG solution (0.5 ng/mL) containing 50 ng/mL of interfering substances were measured by the proposed immunosensor (Fig. 6B). The electrocatalytic current response variation was 4.2%, less than 5% of that without interferences, suggesting the selectivity of the immunosensor is good.

The stability was investigated at 0.05 ng/mL of IgG. As shown in Fig. 6C, the current response of the immunosensor kept constant after 3 days, the current response of the immunosensor only had a minor change of 4.2% after 2 weeks. Subsequently, the current response retained 90.5% of their initial current after 3 weeks. It could be found that current responses to same concentration of IgG has no apparent change compared to the immunosensor freshly prepared which was used to directly detect the same concentration of IgG without being stored, suggesting the stability of the immunosensors was also acceptable. The better stability can be attributed to the good biocompatibility of transducing materials. The reproducibility, selectivity and stability were all acceptable, indicating that the proposed immunosensor was suitable for quantitative detection of IgG in real samples.

### Real sample analysis

In order to test the feasibility and precision of the proposed label-free immunosensor, standard addition method was used to detect the recoveries of different concentrations of IgG in human serum samples. As shown in Supplementary Table S2, the RSD was in the range from 1.40% to 3.01% and the recovery was in the range from 99.53% to 100.4%.The results imply that the proposed immunosensor could be effectively applied to the quantitative determination of IgG in human serums.

## Conclusions

In this paper, a novel and ultrasensitive label-free electrochemical immunosensor for quantitative detection of IgG was prepared using Au@Ag/GS-Fe_3_O_4_/Cd^2+^ as a signal amplification platform. To ensure a high-performance electrochemical immunosensor, Au@Ag/GS-Fe_3_O_4_/Cd^2+^ was immobilized on the electrode, which can improve the electronic transmission rate and increase the surface area to capture a larger amount of antibodies. The proposed label-free immunosensor displayed wide linear range, low detection limit, high sensitivity, good reproducibility, long-term stability and acceptable selectivity. Herein, this proposed method will not only expand the application of Au@Ag/GS-Fe_3_O_4_/Cd^2+^, but also provide an attractive way to detect other biomolecules.

## Additional Information

**How to cite this article**: Li, F. *et al.* An ultrasensitive label-free electrochemical immunosensor based on signal amplification strategy of multifunctional magnetic graphene loaded with cadmium ions. *Sci. Rep.* **6**, 21281; doi: 10.1038/srep21281 (2016).

## Supplementary Material

Supplementary Information
