# Supplementary material for: An ultrasensitive label-free electrochemical immunosensor based on signal amplification strategy of multifunctional magnetic graphene loaded with cadmium ions
Source: Sci Rep. 2016 Feb 16;6:21281. doi: 10.1038/srep21281 (PMC4754691; doi:10.1038/srep21281)
Supplement: Supplementary Information [file srep21281-s1.pdf]

Supporting Information

An ultrasensitive label-free electrochemical immunosensor based on signal amplification strategy of multifunctional magnetic graphene loaded with cadmium ions

Faying Li[a], Yueyun Li[a,b] *, Yunhui Dong[a], Liping Jiang[a], Ping Wang[a], Qing Liu[a], Hui Liu[a], Qin Wei[b]*

a. School of Chemical Engineering, Shandong University of Technology, Zibo, 255049, P.R. China

b. Key Laboratory of Chemical Sensing & Analysis in Universities of Shandong, School of Chemistry and Chemical Engineering, University of Jinan, Jinan, 250022, P.R. China

*Corresponding author: liyueyun71@163.com; sdjndxwq@163.com

Tel: + 86 533 2781225; fax: + 86 531 2781664 (Yueyun Li).

Tel: + 86 531 82767872; fax: + 86 531 82767367 (Qin Wei).

Table S1 The comparison of different immunosensor for the detection of IgG.

Measurement protocol	Linear range	Limit of detection	References
Electrochemical immunoassay	0.01–200 ng/mL	4 pg/mL	[1]
Sandwich-type electrochemical immunosensor	0.01 pg/mL–100 ng/mL	4.3 fg/mL	[2]
Electrochemiluminescence immunosensor	7.5-100 pg	1.0 pg/mL	[3]
Electrochemical immunosensor	5 pg-50 ng	5.0 pg/mL	[4]
Electrochemical immunosensor	10 fg/mL-100 pg/mL	5.0 fg/mL	[5]
Electrochemical immunoassay	0.01-10.0 ng/mL	6.9 pg/mL	[6]
Electrochemical immunosensor	5 fg/mL-50 ng/mL	2 fg/mL	This work

Table S2 Determination of IgG in human serum samples with the proposed immunosensor.

**Initial concentration (ng/mL)**	**Added concentration (ng/mL)**	**Measured concentration (ng/mL)**	**Average value (ng/mL)**	**RSD (%,n=5)**	**Recovery (%, n=5)**
1.01	1.00	1.97, 2.05, 2.09, 1.94, 2.03	2.016	3.01	100.3
1.01	2.00	2.93, 2.95, 3.01, 3.06, 3.03	2.996	1.822	99.53
1.01	4.00	4.99, 5.02, 5.09, 5.11, 4.94	5.03	1.40	100.4

References

1. Cao, X., Liu, S., Feng, Q. & Wang, N. Silver nanowire-based electrochemical immunoassay for sensing immunoglobulin G with signal amplification using strawberry-like ZnO nanostructures as labels. *Biosens. Bioelectron.* **49**, 256-262 (2013).
2. Zhang, S. et al. Copper-doped titanium dioxide nanoparticles as dual-functional labels for fabrication of electrochemical immunosensors. *Biosens. Bioelectron.* **59**, 335-341 (2014).
3. Tian, D., Duan, C., Wang, W. & Cui, H. Ultrasensitive electrochemiluminescence immunosensor based on luminol functionalized gold nanoparticle labeling. *Biosens. Bioelectron.* **25**, 2290-2295 (2010).
4. Zhang, J., Pearce, M.C., Ting, B.P. & Ying, J.Y. Ultrasensitive electrochemical immunosensor employing glucose oxidase catalyzed deposition of gold nanoparticles for signal amplification. *Biosens. Bioelectron.* **27**, 53-57 (2011).
5. Ding, Y. et al. A water-dispersible, ferrocene-tagged peptide nanowire for amplified

electrochemical immunosensing. *Biosens. Bioelectron.* **48**, 281-286 (2013).

6. Lai, G., Zhang, H., Yong, J. & Yu, A. In situ deposition of gold nanoparticles on polydopamine functionalized silica nanosphere for ultrasensitive nonenzymatic electrochemical immunoassay. *Biosens. Bioelectron.* **47**, 178-183 (2013).
